# Common laboratory blood test immune panel markers are useful for grading ulcerative colitis endoscopic severity

**DOI:** 10.1186/s12876-022-02634-x

**Published:** 2022-12-26

**Authors:** Jiawei Cui, Xiujuan Li, Zhiqiang Zhang, Hongliang Gao, Jian Li

**Affiliations:** 1 The Second Department of Gastroenterology, The First Affiliated Hospital of Xinjiang Medical University, Urumqi, 830011 Xinjiang China; 2 Department of Pathophysiology, College of Basic Medical, Xinjiang Medical University, Urumqi, 830011 China

**Keywords:** Ulcerative colitis, Endoscopic activity, Extensive colitis, C-reactive protein, CBC, Laboratory test

## Abstract

**Background:**

At present, many indicators reflect the clinical disease activity of ulcerative colitis (UC). However, commonly used inflammatory markers do not show good utility for indicating endoscopic disease activity. The purpose of this study was to evaluate high sensitivity C-reactive protein (hs-CRP), C-reactive protein to albumin ratio (CAR), inflammatory markers, and complete blood count (CBC) related parameters in patients with UC as simple, non-invasive, and independent markers of endoscopic activity (EA).

**Methods:**

We retrospectively collected extensive data from the hospital medical records of 386 patients who presented with UC to the First Affiliated Hospital of Xinjiang Medical University (Urumqi, China) from 2018 to 2022 January. The Mayo endoscopic score (MES) was used to evaluate endoscopic disease activity. All included patients were defined as the MES-All group; those with extensive colitis (E3) were defined as the MES-E3 group. Demographics, laboratory parameters, endoscopic results, the extent of disease, and drug history were recorded and analyzed.

**Results:**

For patients in the MES-All or MES-E3 group, hs-CRP, CAR, neutrophil to lymphocyte ratio (NLR), and platelet to lymphocyte ratio (PLR) were significantly higher in EA UC patients than in those with mucosal healing. The mean platelet volume (MPV) and lymphocyte to monocyte ratio were significantly lower in active disease than in the patient’s remission (*p* < 0.001). ROC analysis showed that in the MES-All and MES-E3 groups, the cutoff values of hs-CRP activity under endoscopy were 5.32 mg/L (AUC 0.850, sensitivity 77.6%, specificity 81.9%) and 5.16 mg/L (AUC 0.902, sensitivity 86.9%, specificity 85.4%) respectively, and the cutoff values of CAR were 0.14 (AUC 0.853, sensitivity 76.8%, specificity 84.8%) and 0.18 (AUC 0.904, sensitivity 81.8%, specificity 89.6%) respectively. Multivariate logistic regression analysis showed that hs-CRP, CAR, NLR, and PLR identified UC EA, while decreased MPV reflected inflammatory activity in the UC mucosa.

**Conclusion:**

Especially in patients with extensive colitis, hs-CRP and CAR are closely related to EA and show a higher diagnostic value compared to the related CBC parameters. The aforementioned indicators are simple and non-invasive independent markers that reflect the EA in UC.

**Supplementary Information:**

The online version contains supplementary material available at 10.1186/s12876-022-02634-x.

## Background

Ulcerative colitis (UC) is a chronic nonspecific inflammatory disease, that is characterized by a process of recurrence and remission. With treatment endpoints that target "mucosal healing (MH)" rather than mere "remission", comes a growing need to improve diagnostic and monitoring tools. At present, the diagnosis and monitoring of UC, monitoring of responses to intervention, and the detection of MH depend on endoscopy. However, colonoscopy is invasive, expensive, and requires intestinal preparation. In addition, at least one-third of UC patients in the remission stage have gastrointestinal symptoms such as abdominal pain and diarrhea without any signs of disease activity under endoscopy [1]. Studies have found that previous inflammatory UC bouts lead to residual visceral hypersensitivity; even if the inflammatory infiltration subsides and the intestinal structure recovers, visceral hypersensitivity persists [2]. Such patients have repeated symptoms but no identifiable inflammatory activity in the intestinal tract [1]. Repeat endoscopy is not appropriate due to its invasiveness, expense, and other shortcomings, including poor acceptance by patients [3]. Simple markers that reflect endoscopic findings are needed to predict the "active period" and "remission period" of the disease to help determine whether the patient has entered a "relapse". In particular, non-invasive indicators are desired because of their convenience, repeatability, objectivity, and patient comfort.

Blood parameters such as C-reactive protein (CRP), erythrocyte sedimentation rate (ESR), hemoglobin (Hb), white blood cell count (WBC), and albumin (ALB) are markers that are commonly used to judge disease activity [4]. However, UC inflammation is limited to the intestinal mucosa and has a low correlation with systemic disease activity, so its quantification is not particularly helpful in the diagnosis of UC, especially as a correlate of endoscopic activity (EA) [5]. Fecal calprotectin has high accuracy in measuring intestinal inflammation. However, due to high cost, long time requirement, and the influence of intestinal movement, it is still not commonly used in clinical practice in most areas. In addition, it requires the collection of fecal sample which is cumbersome and has poor patient acceptability [6, 7]. High sensitivity C-reactive protein (hs-CRP) has attracted interest in UC in recent years due to its high sensitivity and accuracy, and its relationship with the clinical and even EA of inflammatory bowel disease (IBD) [8]. It has been found that hs-CRP can better reflect the inflammation of the colonic mucosa than fecal calprotectin, and can reliably identify pancolitis [9]. In addition, CRP also shows good diagnostic efficacy in evaluating acute severe UC, and can replace ESR as a classic indicator in the Truelove and Witts criteria [10]. C-reactive protein to albumin ratio (CAR), as a CRP-derived indicator, has shown good diagnostic efficacy in judging disease activity. A correlation was reported between CAR and Crohn's disease activity [11]. Gibson et al. [12] reported that CAR on day 3 in UC is more accurate than CRP on day 3 or ALB on day 3 in reflecting the hormone response, which is an early predictor of acute hormone -refractory UC. The above research indicates that CRP, a commonly and easily obtained indicator, can be exploited to predict and quantify the development of UC-related inflammation. In recent years, some markers of systemic inflammation that are obtained from the complete blood count (CBC), such as neutrophil to lymphocyte ratio (NLR), platelet to lymphocyte ratio (PLR), and lymphocyte to monocyte ratio (LMR) have been reported as diagnostic and predictive indicators of IBD [13–15].

At present, many indicators reflect the clinical disease activity of UC; however, they show less efficacy as correlates of EA [16]. Therefore, the purpose of our study was to evaluate the relationship between EA and blood inflammatory indicators such as hs-CRP, CAR, NLR, PLR, mean platelet volume (MPV), and LMR in UC patients. We were driven by the desire to reduce the need for invasive endoscopic procedures in individuals with high intestinal sensitivity and no inflammatory activity, while accurately quantifying the extent of UC mucositis before the recurrence of clinical activity to optimize patient management.

## Materials and methods

### Data collection

386 UC patients admitted to the First Affiliated Hospital of Xinjiang Medical University from January 2018 to January 2022 were recruited. We extracted information from medical records, including age, gender, smoking history, body mass index (BMI), course of disease, endoscopic results, extent of disease, and detailed medication history.

*Inclusion criteria* (1) patients presenting for initial and follow-up treatment were diagnosed with UC based on endoscopy, laboratory, radiology, histology, and clinical examination based on the *Chinese consensus on diagnosis and treatment in inflammatory bowel disease (2018, Beijing)* [17]; (2) aged over 18 at the time of the first endoscopy; (3) completed electronic colonoscopy with available laboratory results 3 days before and after the completion of the endoscopy, such as CBC, and inflammatory factors; (4) long term and stable use of hormones, immunosuppressants, and biologics (treatment duration over 3 months without a change in drug dose).

*Exclusion criteria* (1) history of gastrointestinal surgery; (2) other autoimmune diseases; blood system diseases; cancer; or serious medical complications, such as cardiovascular disease and chronic kidney disease; (3) acute intestinal infection (stool cultures with *Clostridium difficile* toxin assay); (4) other systemic infections; (5) long-term and short-term (within 3 months) use of contraceptives, anticoagulants, and antiplatelet aggregation drugs, such as estrogen and progesterone, heparin, NSAIDs, etc.; (6) recent use (within 3 months) of hormones, immunosuppressants, biologics, and other drugs with a dose change.

### Data measurement and evaluation criteria

Blood samples were sent to the Laboratory Test Department of the First Affiliated Hospital of Xinjiang Medical University for examination of leukocyte count, hemoglobin, platelet count, platelet volume, neutrophil count, monocyte count, lymphocyte count, serum albumin, hs-CRP, interleukin-6 (IL-6), and ESR. The blood samples were obtained in the morning on an empty stomach. A routine complete blood count (CBC) analysis was performed on an automatic blood cell analyzer (Beckman-Coulter LH750) following the standard operating procedure recommended by the manufacturer. Hs-CRP was measured using a fluorescence-based immunochromatographic method, namely an I-CHROMA hs-CRP assay (Boditech Med Inc, Gangwondo, Korea), and levels within 0–10 mg/L were regarded as normal. IL-6 was measured using a specific enzyme-linked immunosorbent assay (XIAMEN HUIJIA BIOTECHNOLOGY CO. LTD, China), and levels within 0–7 pg/mL were regarded as normal. Laboratory investigators were blinded to the patient clinical data.

Clinical disease activity was evaluated using Lichtiger’s clinical activity index (CAI) [18]: the presence of diarrhea (number of stools per day), nocturnal diarrhea, visible blood in the stool (percentage of movements), fecal incontinence, abdominal pain or cramping, general well-being, abdominal tenderness, and a need for anti-diarrheal drugs. Clinical activity was defined as a CAI > 3.

Patients were administered oral polyethylene glycol electrolytes one day before the colonoscopy as an intestinal cleaning preparation, and then fasted with water intake allowed. The colonoscopy was conducted by an experienced physician and graded according to the Mayo Endoscopy Score (MES). Patients were divided into one of four grades on a 0–3 point scale: "MH" was defined as 0–1 points, and "EA" was defined as 2–3 points [19]. The extent of UC disease was classified by the extent of colonic involvement according to the Montreal classification as follows [20]: ulcerative proctitis (E1); left-sided colitis (E2); or extensive colitis (E3). The colonoscopy images were read and assessed separately by two experienced doctors blinded to the patient's data and clinical conditions. If the two experts disagreed, the third expert was involved in interpreting the results.

### Statistical analysis

All statistical analyses were conducted using SPSS 22.0 software. The normality of data distribution was tested using the Kolmogorov–Smirnov test. Normally distributed data are shown as mean ± standard deviation (SD) with between-group comparisons made using the Student’s t-test. Non-normally distributed data are represented by median ± interquartile range (IQR) with between-group comparisons made using the Mann–Whitney U test. Categorical data are summarized as counts (%); differences between categorical variables were assessed using the Chi-square test or Fisher's precision probability test. The Kruskal–Wallis H test or a one-way ANOVA was used to compare groups as appropriate, with a Bonferroni correction and post hoc Student–Newman–Keuls (SNK-q) tests. Spearman’s correlation coefficients were used to test between laboratory parameters and MES. We then conducted binary multivariate logistic regression analysis on laboratory indicators to explore independent predictors significantly related to UC EA. Gender, age, BMI, smoking, duration of disease, extent of colonic involvement, 5-aminosalicylic acid, hormone use, immunosuppressants, and biologics were used as confounding factors. To differentiate between EA and MH patients with UC, the optimal cut-off values of CRP, CAR, MPV, NLR, PLR, and LMR with maximum sensitivity and specificity were calculated via receiver operating characteristic (ROC) curve analysis. *p* < 0.05 was considered to be statistically significant.

## Results

### Patients characteristics

386 UC patients were enrolled in this study, with an average age of 45.9 years. Male and female patients accounted for 56.7% and 43.3%, respectively. Patients were divided into “MH” (105 cases) and “EA” (281 cases) based on our criteria. The demographic and clinical characteristics of UC patients are shown in Table 1. Age, sex, BMI, smoking, and duration of disease in remission and active periods were not statistically significant (*p* > 0.05), as shown in Table 2. There was no significant difference in the patient's medication history (5-aminosalicylic acid (5-ASA), glucocorticoids, immunosuppressants, biologics, and no drug treatment) in the remission and the active period (χ^2^ = 3.92, *p* = 0.406). (2 × 3) Fisher's precision probability test was performed on the disease extent. During the remission and active periods, there was a statistical difference between E3 and E1/E2. E3 patients are discussed in detail below (all patients are defined as the MES-All group, and only patients with extensive colitis (E3) are defined as the MES-E3 group).

**Table 1:** General characteristics of patient population

Characteristics	Statistics
Age (year)	45.9 ± 13.6
Male (n, %)	219 (56.7)
BMI (kg/m^2^)	21.99 ± 3.94
Smoke (n, %)	52 (13.5)
Duration (month)	36 (63)
Mayo endoscopic subscore (n, %)	
MES 0	32 (8.3)
MES 1	73 (18.9)
MES 2	164 (42.5)
MES 3	117 (30.3)
Disease extent at baseline (n, %)	
E1	61 (15.9)
E2	79 (20.5)
E3	246 (63.7)
Drug (n, %)	
None	117 (30.3)
5-ASA	247 (64.0)
Glucocorticoid	6 (1.6)
Immunosuppressant	9 (2.3)
Biologics	7 (1.8)
hs-CRP (mg/L)	8.25 (18.71)
CAR	0.216 (0.588)
MPV (fL)	9.70 (1.43)
NLR	2.19 (1.67)
PLR	163.60 (104.71)
LMR	3.52 (2.17)

*BMI* body mass index, *5-ASA* 5-aminosalicylic acid, *hs-CRP* high sensitivity C-reactive protein, *CAR* C reactive protein to albumin ratio, *MPV* mean platelet volume, *NLR* neutrophil to lymphocyte ratio, *LMR* lymphocyte to monocyte ratio, *PLR* platelet to lymphocyte ratio; Values are expressed as mean ± SD, median (IQR), or n (%)

**Table 2:** Comparison of baseline data of UC patients

Parameter	MH	EA	ES	*p* Value
Age (year)	47.4 ± 13.9	45.3 ± 13.5	1.367	0.172
Duration (month)	36 (72)	36 (60)	− 1.059	0.289
BMI (kg/m^2^)	23 (6)	22 (6)	− 1.057	0.291
Gender (F/M)	48/57	119/162	0.353	0.553
Smoking (Y/N)	14/91	38/243	0.002	0.961
Extent (E1/E2/E3)*	30/27/48	31/52/198	20.89	< 0.001
Drug*				
None	27	90	3.92	0.406
5-ASA	74	173		
Glucocorticoid	0	6		
Immunosuppressant	2	7		
Biologics	2	5		

*UC* ulcerative colitis, *ES* effect size, *5-ASA* 5-aminosalicylic acid, *MH* mucosal healing, *EA* endoscopic activity; Values are expressed as mean ± SD or median (IQR); *means Fisher's precision probability test

### Laboratory parameters for grading ulcerative colitis endoscopic severity

Endoscopic severity was divided into four groups according to the MES. There was no significant difference in age, sex, smoking, BMI, or duration of disease between the active and inactive groups, in the MES-All or MES-E3 groups (*p* > 0.05). In the MES-All group, the levels of hs-CRP, CAR, NLR, PLR, LMR, and MPV were significantly different among the four subgroups (*p* < 0.001). In the pairwise comparison between groups, the remission group (0–1 points) showed significantly lower hs-CRP, CAR, NLR, and PLR than the activity group (2–3 points); the remission group showed significantly higher LMR and MPV than the activity group, and there was no significant difference in subgroups of the remission group (0–1 points) (Table 3). In the MES-E3 group, the levels of hs-CRP, CAR, NLR, PLR, LMR, and MPV in the four subgroups were also significantly different (*p* < 0.001), as shown in Table 4.

**Table 3:** Intra-group comparisons of laboratory parameters of UC patients in MES-All

Parameters	0–1 Point	2 Point	3 Point	*p* Value	Intra-group		
					1 versus 2	1 versus 3	2 versus 3
Age (years)	47.4 ± 13.9	45.6 ± 13.0	44.8 ± 14.1	0.355			
Gender (F/M)	48/57	72/92	47/70	0.691			
Smoking (Y/N)	14/91	25/139	13/104	0.606			
BMI (kg/m^2^)	22.38 ± 4.27	22.13 ± 3.85	21.45 ± 3.73	0.181			
Duration (month)	36 (72)	36 (69)	36 (60)	0.568			
hs-CRP (mg/L)	2.64 (3.18)	8.42 (8.80)	29.80 (49.22)	< 0.001	< 0.001	< 0.001	< 0.001
IL-6 (pg/ml)	5.69 (5.36)	9.56 (11.02)	26.42 (37.00)	< 0.001	< 0.001	< 0.001	< 0.001
ESR (mm/h)	11.00 (12.00)	24.00 (25.50)	42.00 (30.00)	< 0.001	< 0.001	< 0.001	< 0.001
ALB (g/L)	40.26 (5.81)	39.32 (7.65)	32.69 (9.16)	< 0.001	< 0.001	< 0.001	0.021
CAR	0.06 (0.08)	0.22 (0.26)	0.97 (2.00)	< 0.001	< 0.001	< 0.001	< 0.001
WBC (10^9^/L)	6.03 ± 2.00	7.25 ± 2.72	8.44 ± 3.54	< 0.001	0.002	< 0.001	0.002
Hb (g/L)	135.0 (30.0)	127.0 (36.0)	109.0 (43.0)	< 0.001	< 0.001	< 0.001	0.025
PLT (10^9^/L)	239.6 ± 79.2	306.4 ± 96.3	382.4 ± 106.1	< 0.001	< 0.001	< 0.001	< 0.001
Neutrophil (10^9^/L)	3.24 ± 1.15	4.21 ± 1.63	5.02 ± 1.78	< 0.001	< 0.001	< 0.001	< 0.001
Lymphocyte (10^9^/L)	1.90 (0.82)	1.79 (0.89)	1.68 (0.94)	0.004	0.128	0.003	0.360
Monocyte (10^9^/L)	0.40 (0.19)	0.56 (0.30)	0.68 (0.35)	< 0.001	< 0.001	< 0.001	0.007
MPV (fL)	10.39 ± 1.24	9.70 ± 0.93	9.01 ± 1.14	< 0.001	< 0.001	< 0.001	< 0.001
NLR	1.52 (1.02)	2.20 (1.44)	2.98 (2.08)	< 0.001	< 0.001	< 0.001	< 0.001
LMR	4.77 (2.32)	3.52 (1.78)	2.60 (1.53)	< 0.001	< 0.001	< 0.001	< 0.001
PLR	117.9 (61.4)	161.9 (74.3)	228.1 (128.2)	< 0.001	< 0.001	< 0.001	< 0.001

*UC* ulcerative colitis, *BMI* body mass index, *hs-CRP* high sensitivity C-reactive protein, *IL-6* interleukin 6, *ESR* erythrocyte sedimentation rate, *ALB* albumin, *CAR* C reactive protein to albumin ratio, *WBC* white blood cells, *Hb* hemoglobin, *PLT* platelet, *MPV* mean platelet volume, *NLR* neutrophil to lymphocyte ratio, *LMR* lymphocyte to monocyte ratio, *PLR* platelet to lymphocyte ratio. Values are expressed as mean ± SD or median (IQR). 1 versus 2: 0–1 point group versus 2 ponit group. 1 versus 3: 0–1 point group versus 3 ponit group. 2 versus 3: 2 point group versus 3 ponit group. There is no statistical difference between the 0 point and 1 point group (*p* > 0.05)

**Table 4:** Intra-group comparisons of laboratory parameters of UC patients in MES-E3

Parameters	0–1 point	2 point	3 point	*p* Value	Intra-group		
					1 versus 2	1 versus 3	2 versus 3
Age (years)	47.8 ± 14.3	45.2 ± 14.4	45.5 ± 14.0	0.560			
Gender (F/M)*	21/27	42/56	43/57	0.995			
Smoking (Y/N)*	8/40	18/80	11/89	0.329			
BMI (kg/m^2^)	22.90 ± 4.42	21.55 ± 3.75	21.30 ± 3.76	0.059			
Duration (month)	42.00 (108)	30.00 (76)	30.00 (72)	0.186			
hs-CRP (mg/L)	2.34 (2.60)	10.10 (15.64)	32.20 (61.00)	< 0.001	< 0.001	< 0.001	< 0.001
IL-6 (pg/ml)	5.39 (4.41)	10.68 (16.05)	27.60 (34.64)	< 0.001	< 0.001	< 0.001	< 0.001
ESR (mm/h)	12.50(15.50)	28.00(33.30)	45.50(29.00)	< 0.001	< 0.001	< 0.001	< 0.001
ALB (g/L)	40.49 (5.22)	37.80 (7.43)	31.63 (8.20)	< 0.001	< 0.001	< 0.001	0.007
CAR	0.06 (0.07)	0.27 (0.47)	1.07 (1.97)	< 0.001	< 0.001	< 0.001	< 0.001
WBC (10^9^/L)	5.92 ± 1.87	7.81 ± 3.07	8.55 ± 3.69	< 0.001	0.002	< 0.001	0.300
Hb (g/L)	139.5 (34.0)	124.0 (32.0)	106.5 (38.0)	< 0.001	< 0.001	< 0.001	0.082
PLT (10^9^/L)	236.4 ± 82.2	314.5 ± 102.9	391.0 ± 106.6	< 0.001	< 0.001	< 0.001	< 0.001
Neutrophil (10^9^/L)	3.28 ± 1.19	4.53 ± 1.79	5.09 ± 1.82	< 0.001	< 0.001	< 0.001	0.066
Lymphocyte (10^9^/L)	1.87 (0.93)	1.86 (0.74)	1.68 (0.97)	0.021	1.000	0.059	0.061
Monocyte (10^9^/L)	0.40 (0.18)	0.60 (0.35)	0.69 (0.36)	< 0.001	< 0.001	< 0.001	0.526
MPV (fL)	10.69 ± 1.18	9.50 ± 0.95	8.94 ± 1.16	< 0.001	< 0.001	< 0.001	< 0.001
NLR	1.50 (1.33)	2.26 (1.65)	3.01 (2.00)	< 0.001	0.001	< 0.001	0.003
LMR	4.86 (3.00)	3.30 (1.93)	2.59 (1.54)	< 0.001	< 0.001	< 0.001	0.013
PLR	122.1 (69.0)	161.2 (66.8)	231.8 (120.5)	< 0.001	0.001	< 0.001	< 0.001

*UC* ulcerative colitis, *BMI* body mass index, *hs-CRP* high sensitivity C-reactive protein, *IL-6* interleukin 6, *ESR* erythrocyte sedimentation rate, *ALB* albumin, *CAR* C reactive protein to albumin ratio, *WBC* white blood cells, *Hb* hemoglobin, *PLT* platelet, *MPV* mean platelet volume, *NLR* neutrophil to lymphocyte ratio, *LMR* lymphocyte to monocyte ratio, *PLR* platelet to lymphocyte ratio. Values are expressed as mean ± SD or median (IQR). 1 versus 2: 0–1 point group versus 2 ponit group. 1 versus 3: 0–1 point group versus 3 ponit group. 2 versus 3: 2 point group versus 3 ponit group. There is no statistical difference between the 0 point and 1 point group (*p* > 0.05)

### Correlation between the MES and laboratory parameters

**Fig. 1:**
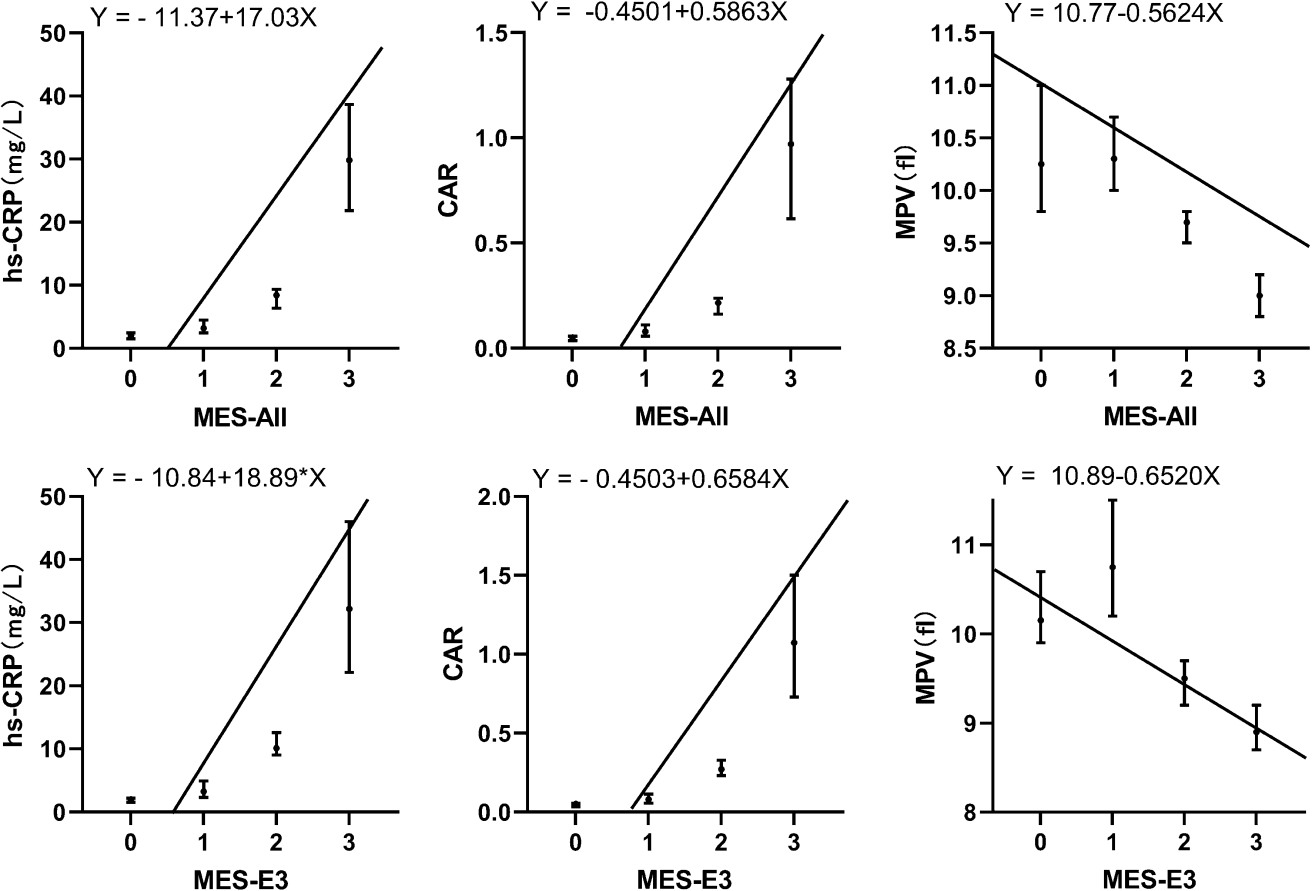
Correlations between UC endoscopic activity and laboratory parameters

As shown in Table 5, Spearman correlation analyses show the correlation between laboratory parameters and EA. In the MES-All group, hs-CRP (r = 0.667, *p* < 0.001), CAR (r = 0.678, *p* < 0.001), NLR (r = 0.431, *p* < 0.001), and PLR (r = 0.520, *p* < 0.001) were positively correlated with EA, while MPV (r =  − 0.448, *p* < 0.001) and LMR (r = − 0.477, *p* < 0.001) were negatively correlated with EA. Hs-CRP, CAR, and MPV linear regression equation and Interval charts with medians (P25, P75) are shown in Fig. 1. In addition, the above indices were also significantly correlated with the EA of patients in the MES-E3 group (*p* < 0.001).

**Table 5:** Spearman’s correlation coefficients between endoscopic activity and laboratory parameters

Variables	MES-All		MES-E3	
	Rho	*p* Value	Rho	*p* Value
hs-CRP	0.667	< 0.001	0.645	< 0.001
IL-6	0.496	< 0.001	0.501	< 0.001
ESR	0.527	< 0.001	0.507	< 0.001
PLT	0.501	< 0.001	0.502	< 0.001
MPV	− 0.448	< 0.001	− 0.488	< 0.001
NLR	0.431	< 0.001	0.398	< 0.001
LMR	− 0.477	< 0.001	− 0.428	< 0.001
PLR	0.520	< 0.001	0.523	< 0.001
CAR	0.678	< 0.001	0.663	< 0.001

*hs-CRP* high sensitivity C-reactive protein, *IL-6* interleukin 6, *ESR* erythrocyte sedimentation rate, *CAR* C reactive protein to albumin ratio, *PLT* platelet, *MPV* mean platelet volume, *NLR* neutrophil to lymphocyte ratio, *LMR* lymphocyte to monocyte ratio, *PLR* platelet to lymphocyte ratio

### Multivariate logistic regression analyses

The specific values of hs-CRP, CAR, NLR, PLR, MPV, and LMR and their relationships with the activity period are shown in Table 6, based on binary multivariate logistic regression analyses. After adjusting for the interference of confounding factors in the MES-All group, hs-CRP (OR 1.25, 95% CI 1.16–1.35), CAR (OR 2.23, 95% CI 1.70–2.94), NLR (OR 2.43, 95% CI 1.79–3.30), and PLR (OR 1.02, 95% CI 1.01–1.02) were found to be significantly related to an increased risk of EA, while increased MPV (OR 0.48, 95% CI 0.38–0.61) indicated the remission of EA. In the MES-E3 group, the correlation strength between the above indices and EA increased significantly, especially CAR (OR 3.48, 95% CI 2.16–5.61). Detailed displays of variables and confounding factors are shown in  supplementary material (Additional file 1).

**Table 6:** Multivariate logistic regression analysis results of laboratory parameters

Variable	MES-ALL		MES-E3	
	OR (95%CI)	Adjusted OR (95%CI)	OR (95%CI)	Adjusted OR (95%CI)
hs-CRP	1.25 (1.16, 1.33)	1.25 (1.16, 1.35)	1.35 (1.21, 1.51)	1.43 (1.25, 1.64)
MPV	0.46 (0.37, 0.58)	0.48 (0.38, 0.61)	0.32 (0.22, 0.46)	0.31 (0.21, 0.46)
NLR	2.42 (1.82, 3.22)	2.43 (1.79, 3.30)	2.38 (1.63, 3.47)	2.69 (1.74, 4.16)
LMR	1.00 (0.99, 1.01)	1.00 (0.99, 1.01)	0.62 (0.51, 0.75)	0.56 (0.45, 0.70)
PLR	1.02 (1.01, 1.02)	1.02 (1.01, 1.02)	1.02 (1.01, 1.02)	1.02 (1.01, 1.03)
CAR	2.20 (1.70, 2.85)	2.23 (1.70, 2.94)	2.90 (1.93, 4.35)	3.48 (2.16, 5.61)

*OR* odds ratio, *CI* confidence interval; Gender (female), age, BMI, smoking (no), duration of disease, extent of colon (E3), 5-aminosalicylic acid (no), glucocorticoid (no), immunosuppressants (no), and biologics (no) were used as confounding factors, the categories analyzed are described with the reference category displayed in brackets

### Model performance

**Fig. 2:**
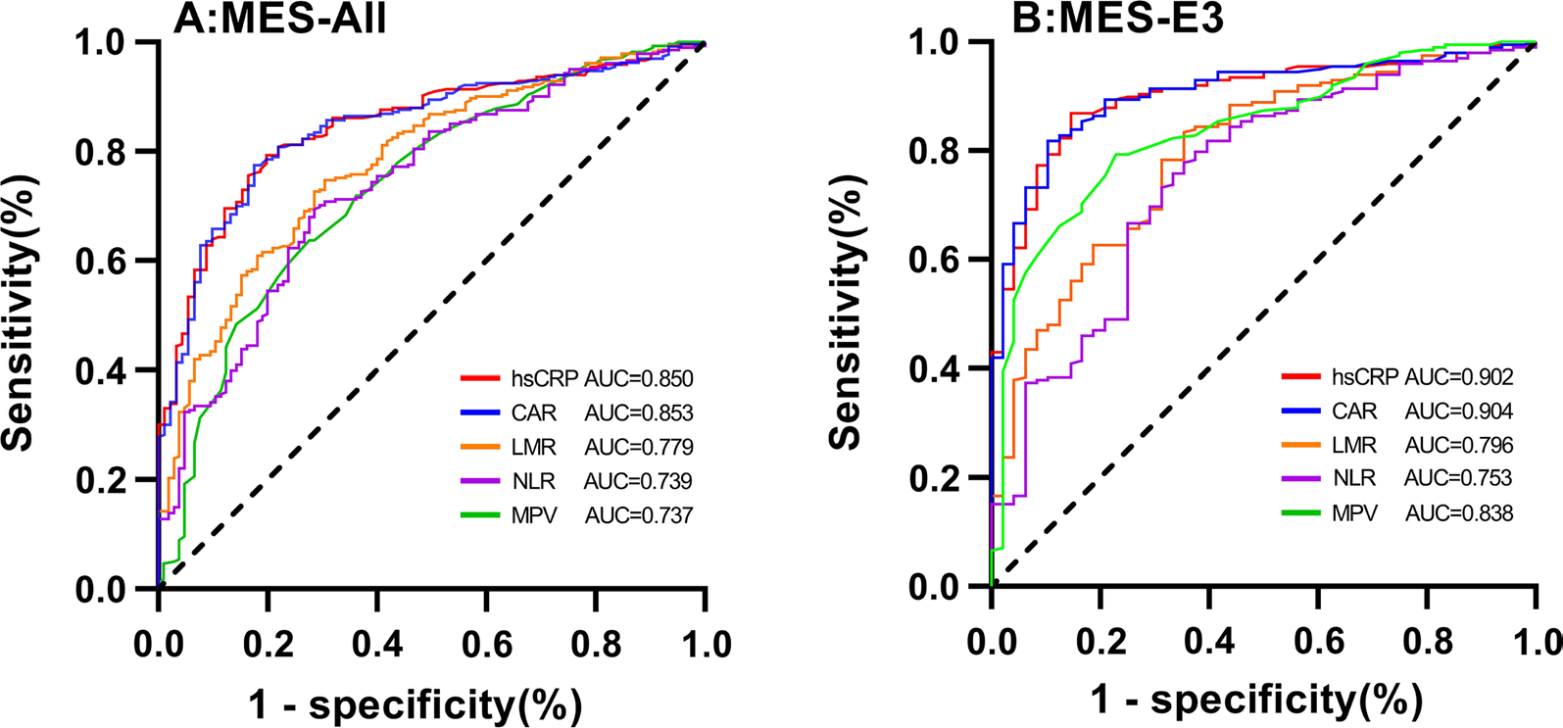
The receiver operating characteristic curve (ROC) of laboratory parameters in predicting endoscopic activity in ulcerative colitis. **A** ROC curve endoscopic activity of all patients. **B** ROC curve endoscopic activity of E3 patients

The predictive value of the laboratory indicators for UC activity and the remission period was tested by ROC curve analysis (Table 7). As shown in Fig. 2, the ROC curves describe the laboratory parameter values relative to the activity period. In the MES-All group, ROC analyses showed that the AUC value of hs-CRP was 0.850 (sensitivity 77.6%, specificity 81.9%) and the AUC of CAR was 0.853 (sensitivity 76.8%, specificity 84.8%); these were higher than other laboratory indicators. In the MES-E3 group, the hs-CRP AUC was 0.902 (sensitivity 86.9%, specificity 85.4%), CAR AUC was 0.904 (sensitivity 81.8%, specificity 89.6%), and the MPV AUC was 0.838 (sensitivity 77.1%, specificity 79.3%). Compared with the MES-All group, the above three indicators had significant improvement in AUC, sensitivity, and specificity.

**Table 7:** Diagnostic value of laboratory parameters in endoscopic activity

Group	Variables	AUCs	SE	95% CI	Cut-offs	Sensitivity (%)	Specificity (%)
MES-All	hs-CRP	0.850	0.020	0.810–0.890	5.32	77.6	81.9
	MPV	0.737	0.029	0.681–0.793	9.72	72.4	63.7
	NLR	0.739	0.028	0.685–0.794	1.97	69.4	71.4
	LMR	0.779	0.025	0.730–0.829	3.92	69.5	71.4
	PLR	0.779	0.026	0.729–0.830	145.66	75.1	70.5
	CAR	0.853	0.020	0.814–0.892	0.14	76.8	84.8
MES-E3	hs-CRP	0.902	0.022	0.859–0.944	5.16	86.9	85.4
	MPV	0.838	0.030	0.780–0.897	9.95	77.1	79.3
	NLR	0.753	0.040	0.675–0.830	1.87	75.8	66.7
	LMR	0.796	0.035	0.728–0.864	4.32	64.6	83.3
	PLR	0.790	0.036	0.720–0.860	160.28	67.2	79.2
	CAR	0.904	0.021	0.863–0.946	0.18	81.8	89.6

*AUC* area under curve, *SE* standard error, *CI* confidence interval, *hs-CRP* high sensitivity C-reactive protein, *CAR* C reactive protein to albumin ratio, *MPV* mean platelet volume, *NLR* neutrophil to lymphocyte ratio, *LMR* lymphocyte to monocyte ratio, *PLR* platelet to lymphocyte ratio

## Discussion

The purpose of this study was to explore the diagnostic value of inflammatory markers such as hs-CRP, CAR, NLR, PLR, MPV, and LMR in evaluating the endoscopic activity of UC. Compared with MH, the active group showed higher hs-CRP, CAR, PLT, NLR, and PLR, while the MPV and LMR were decreased significantly in all patients. These parameters are closely related to UC endoscopic activity. In ROC analysis, hs-CRP and CAR showed good diagnostic value in endoscopic grading, without a significant difference in their AUCs (*p* > 0.05). After adjusting for the influence of confounding factors, we found that hs-CRP, CAR, NLR, and PLR may be independent predictors of UC EA. In addition, we found that MPV decrease may be an indicator of increased UC disease activity.

Because of the high proportion of patients with pancolitis (E3) in this study, we isolated E3 patients from medical records for correlation analysis. Using both Spearman correlation analysis and logistic regression analysis, we found that the relationship between the above indicators and EA was significantly strengthened. To our surprise, compared with the MES-All group, the area under the curve for hs-CRP, CAR, and MPV in the MES-E3 group was significantly greater, suggesting excellent diagnostic value in this group. Thus, based on our findings, for E3 patients the hs-CRP, CAR, and MPV laboratory parameters can be used to determine whether the mucosa is inflamed and help guide decision-making on subsequent endoscopic interventions.

Blood composition analysis is a simple and cheap method to evaluate the activity of UC disease. In patients with IBD, the relationship between parameters related to CBC and disease activity has been confirmed by some studies [21–23]. These studies showed that NLR and PLR are increased while LMR is decreased in patients with active IBD. In our study, although we confirmed that NLR and PLR are significantly related to endoscopic activity, their diagnostic efficacy in predicting endoscopic activity was not satisfactory. The use of the Truelove Witts severity index (MTWSI), Mayo score, and other criteria to evaluate clinical activities when evaluating the diagnostic efficacy of parameters in the above studies is subjective and can be affected by symptoms and physician evaluation [24]. In our work, we directly evaluated endoscopic activity which is the gold standard approach for evaluating mucosal status; the difference in findings between endoscopic examination and clinical assessments may be one of the main sources of differences between previous studies and our work. In addition, different sample sizes or methodological disparities can also lead to different results. We found that the LMR decreased compared with the active period and was significantly negatively correlated with disease activity. However, it was not an independent predictor of endoscopic activity in multivariate logistic regression analysis (*p* > 0.05). Logistic regression was not conducted by Xu et al. other studies [23, 25], and sample sizes were small, so the diagnostic value of LMR in the evaluation of mucosal activity by UC endoscopy needs to be further evaluated.

In the pathological state, MPV is related to platelet activity [26]. Research shows that large platelets have more cell particles, show higher expression of adhesion molecules, and can be activated faster, which will lead to excessive platelet activity and increase the risk of clot formation [27]. At the same time, these cells migrate rapidly to sites of inflammation, where they will be activated and consumed, which may explain the decline of MPV in patients with persistent inflammation [28]. In our study, we demonstrated that the MPV was negatively correlated with the endoscopic activity index. This is similar to previous studies [29–31]; low levels of MPV may indicate mucosal inflammatory activity in UC.

Among the non-invasive markers, CRP is widely used to assess the presence of acute or chronic inflammation. However, compared with the permeability inflammation of CD, UC inflammation is limited to the intestinal mucosa and has a low correlation with systemic activity [5]. Therefore, its performance is lower in UC. At critical values of 0.3 or 0.5 mg/dL, its sensitivity and specificity are poor [32]. In recent years, the sensitivity of CRP detection has been improved [8]. Hs-CRP can detect values below 0.3 mg/dL and is related to the clinical and endoscopic activity of UC [8]. In the MES group and MES-E3 groups, the cutoff values of hs-CRP for endoscopic activity were 5.32 mg/L (AUC 0.850, sensitivity 77.6%, specificity 81.9%) and 5.16 mg/L (AUC 0.902, sensitivity 86.9%, specificity 85.4%), respectively, showing good diagnostic efficacy in evaluating endoscopic activity. The CAR, derived from CRP, was initially used as a new predictor to identify critical patients. Few previous studies have evaluated CAR in IBD patients. One study showed a correlation between CAR and Crohn's disease activity [11]. In a study by Chen et al. [33], CAR (AUC 0.925, sensitivity 75.8%, specificity 92.0%) was found to be an independent predictor of UC and CD disease to distinguish between remission and active periods. In our study, the diagnostic efficacy of CAR (MES group: AUC 0.853, sensitivity 76.8%, specificity 84.8%; MES-E3 group: AUC 0.904, sensitivity 81.8%, specificity 89.6%) was similar to that of hs-CRP, showing a good predictive value for endoscopic activity in E3 patients.

## Conclusion

In conclusion, hs-CRP and CAR are closely related to the activity of UC endoscopic grading in UC patients. After adjusting for the influence of confounding factors, hs-CRP, CAR, NLR, and PLR may be effective markers to distinguish the endoscopic activity of UC. Among them, hs-CRP and CAR reflect the mucosal activity of UC endoscopy, while an increase in MPV may indicate the reduction of UC mucosal inflammation. When selecting the appropriate cut-off values to determine disease activity, especially the pancolitis group, hs-CRP and CAR showed higher classification and diagnostic values compared to CBC parameters. As such, they offer simple, economic, and effective predictors of UC endoscopic activity. According to this study, the above indicators can be used to measure UC mucosal activity, but validation through the subsequent, large-sample, multi-center cohort studies is necessary. By identifying these factors in the clinic, we can reduce some of the risks associated with endoscopy, adjust treatment strategies, provide more individualized maintenance treatments before patients show symptoms of clinical activity, and block the progress of the disease, these approaches are beneficial to patients who require interventions and those with inactive UC who can be allowed to discontinue medication usage.

## Supplementary Information

**Additional file 1.** Detailed display of the relationship between various indicators and confounding factors.

## Data Availability

The datasets used and/or analyzed during the current study are available from the corresponding author on reasonable request.
