# Supplementary material for: Common laboratory blood test immune panel markers are useful for grading ulcerative colitis endoscopic severity
Source: BMC Gastroenterol. 2022 Dec 26;22:540. doi: 10.1186/s12876-022-02634-x (PMC9791766; doi:10.1186/s12876-022-02634-x)
Supplement: Supplementary file 1 — Additional file 1. Detailed display of the relationship between various indicators and confounding factors. [file 12876_2022_2634_MOESM1_ESM.pdf]

## Additional file 1: Detailed display of the relationship between various indicators and confounding factors

Additional table 1. Binary multivariate logistic regression analyses of hs-CRP and confounding factors in MES-ALL

| Variable | B | SEE | Wald | P value | OR | 95%CI |
|---|---|---|---|---|---|---|
| Age | -0.012 | 0.011 | 1.153 | 0.283 | 0.988 | 0.968-1.010 |
| Gender (female) | -0.038 | 0.321 | 0.014 | 0.907 | 0.963 | 0.513-1.806 |
| Smoke (no) | -0.275 | 0.429 | 0.411 | 0.521 | 0.759 | 0.328-1.761 |
| BMI | -0.019 | 0.038 | 0.259 | 0.611 | 0.981 | 0.911-1.056 |
| Duration | -0.003 | 0.002 | 2.101 | 0.147 | 0.997 | 0.993-1.001 |
| Extent (E3) | -0.278 | 0.276 | 1.014 | 0.314 | 0.757 | 0.440-1.301 |
| 5-ASA (no) | 0.195 | 0.309 | 0.399 | 0.528 | 1.215 | 0.664-2.226 |
| Glucocorticoid (no) | -19.425 | 12548.962 | 0.000 | 0.999 | 0.000 | - |
| immunosuppressants (no) | 0.125 | 0.987 | 0.016 | 0.899 | 1.133 | 0.164-7.844 |
| biologics (no) | 0.316 | 0.954 | 0.110 | 0.741 | 1.371 | 0.211-8.893 |
| Hs-CRP | 0.224 | 0.038 | 35.452 | 0.000 | 1.251 | 1.162-1.347 |

B: regression coefficient; SEE: standard error of estimate; OR: odds ratio; CI: confidence interval; BMI: body mass index; 5-ASA: 5-aminosalicylic acid; hs-CRP: high sensitivity C-reactive protein; gender (female), age, BMI, smoking (no), duration of disease, extent of colon (E3), 5-aminosalicylic acid (no), glucocorticoid (no), immunosuppressants (no), and biologics (no) were used as confounding factors, the categories analyzed are described with the reference category displayed in brackets.

Additional table 2. Binary multivariate logistic regression analyses of MPV and confounding factors in MES-ALL

| Variable | B | SEE | Wald | P value | OR | 95%CI |
|---|---|---|---|---|---|---|
| Age | -0.009 | 0.010 | 0.883 | 0.347 | 0.991 | 0.971-1.010 |
| Gender (female) | 0.177 | 0.294 | 0.364 | 0.547 | 1.194 | 0.671-2.122 |
| Smoke (no) | -0.217 | 0.393 | 0.306 | 0.580 | 0.805 | 0.373-1.737 |
| BMI | -0.007 | 0.036 | 0.038 | 0.846 | 0.993 | 0.926-1.065 |
| Duration | -0.002 | 0.002 | 1.397 | 0.237 | 0.998 | 0.994-1.002 |
| Extent (E3) | -0.876 | 0.258 | 11.508 | 0.001 | 0.416 | 0.251-0.691 |
| 5-ASA (no) | 0.465 | 0.293 | 2.509 | 0.113 | 1.591 | 0.896-2.828 |
| Glucocorticoid (no) | -19.822 | 15973.780 | 0.000 | 0.999 | 0.000 | - |
| immunosuppressants (no) | -0.102 | 0.944 | 0.012 | 0.914 | 0.903 | 0.142-5.739 |
| biologics (no) | 0.255 | 0.898 | 0.081 | 0.776 | 1.291 | 0.222-7.499 |
| MPV | -0.734 | 0.123 | 35.740 | 0.000 | 0.480 | 0.377-0.610 |

B: regression coefficient; SEE: standard error of estimate; OR: odds ratio; CI: confidence interval; BMI: body mass index; 5-ASA: 5-aminosalicylic acid; MPV: mean platelet volume; gender (female), age, BMI, smoking (no), duration of disease, extent of colon (E3), 5-aminosalicylic acid (no), glucocorticoid (no), immunosuppressants (no), and biologics (no) were used as confounding factors, the categories analyzed are described with the reference

category displayed in brackets.

Additional table 3. Binary multivariate logistic regression analyses of NLR and confounding factors in MES-ALL

| Variable | B | SEE | Wald | P value | OR | 95%CI |
|---|---|---|---|---|---|---|
| Age | -0.006 | 0.010 | 0.397 | 0.528 | 0.994 | 0.975-1.013 |
| Gender (female) | 0.068 | 0.290 | 0.055 | 0.814 | 1.070 | 0.607-1.889 |
| Smoke (no) | -0.233 | 0.407 | 0.326 | 0.568 | 0.792 | 0.357-1.761 |
| BMI | -0.027 | 0.035 | 0.576 | 0.448 | 0.974 | 0.909-1.043 |
| Duration | -0.004 | 0.002 | 4.693 | 0.030 | 0.996 | 0.992-1.000 |
| Extent (E3) | -0.792 | 0.259 | 9.322 | 0.002 | 0.453 | 0.273-0.753 |
| 5-ASA (no) | 0.493 | 0.290 | 2.900 | 0.089 | 1.638 | 0.928-2.889 |
| Glucocorticoid (no) | -19.719 | 14509.032 | 0.000 | 0.999 | 0.000 | - |
| immunosuppressants (no) | 0.639 | 0.920 | 0.483 | 0.487 | 1.895 | 0.312-11.495 |
| biologics (no) | 0.085 | 0.904 | 0.009 | 0.925 | 1.089 | 0.185-6.399 |
| NLR | 0.888 | 0.157 | 31.977 | 0.000 | 2.429 | 1.786-3.304 |

B: regression coefficient; SEE: standard error of estimate; OR: odds ratio; CI: confidence interval; BMI: body mass index; 5-ASA: 5-aminosalicylic acid; NLR: neutrophil to lymphocyte ratio; gender (female), age, BMI, smoking (no), duration of disease, extent of colon (E3), 5-aminosalicylic acid (no), glucocorticoid (no), immunosuppressants (no), and biologics (no) were used as confounding factors, the categories analyzed are described with the reference category displayed in brackets.

Additional table 4. Binary multivariate logistic regression analyses of LMR and confounding factors in MES-ALL

| Variable | B | SEE | Wald | P value | OR | 95%CI |
|---|---|---|---|---|---|---|
| Age | -0.010 | 0.009 | 1.086 | 0.297 | 0.990 | 0.973-1.009 |
| Gender (female) | 0.312 | 0.273 | 1.303 | 0.254 | 1.366 | 0.800-2.334 |
| Smoke (no) | -.226 | 0.373 | 0.367 | 0.545 | 0.798 | 0.384-1.658 |
| BMI | -0.031 | 0.034 | 0.848 | 0.357 | 0.970 | 0.908-1.036 |
| Duration | -0.003 | 0.002 | 2.799 | 0.094 | 0.997 | 0.994-1.001 |
| Extent (E3) | -1.095 | 0.244 | 2.103 | 0.000 | 0.335 | 0.207-0.540 |
| 5-ASA (no) | 0.352 | 0.272 | 1.667 | 0.197 | 1.422 | 0.833-2.425 |
| Glucocorticoid (no) | -2.395 | 15806.025 | 0.000 | 0.999 | 0.000 | - |
| immunosuppressants (no) | 0.102 | 0.855 | 0.014 | 0.905 | 1.107 | 0.207-5.911 |
| biologics (no) | 0.340 | 0.880 | 0.149 | 0.699 | 1.405 | 0.250-7.880 |
| LMR | 0.003 | 0.006 | 0.247 | 0.619 | 1.003 | 0.992-1.014 |

B: regression coefficient; SEE: standard error of estimate; OR: odds ratio; CI: confidence interval; BMI: body mass index; 5-ASA: 5-aminosalicylic acid; LMR: lymphocyte to monocyte ratio; gender (female), age, BMI, smoking (no), duration of disease, extent of colon (E3), 5-aminosalicylic acid (no), glucocorticoid (no), immunosuppressants (no), and biologics (no) were used as confounding factors, the categories analyzed are described with the reference category displayed in brackets.

Additional table 5. Binary multivariate logistic regression analyses of PLR and confounding factors in MES-ALL

| Variable | B | SEE | Wald | P value | OR | 95%CI |
|---|---|---|---|---|---|---|
| Age | 0.001 | 0.010 | 0.013 | 0.908 | 1.001 | 0.981-1.021 |
| Gender (female) | 0.562 | 0.309 | 3.319 | 0.068 | 1.755 | 0.958-3.214 |
| Smoke (no) | -0.111 | 0.405 | 0.075 | 0.784 | 0.895 | 0.404-1.980 |
| BMI | -0.026 | 0.036 | 0.528 | 0.467 | 0.974 | 0.907-1.046 |
| Duration | -0.003 | 0.002 | 2.608 | 0.106 | 0.997 | 0.993-1.001 |
| Extent (E3) | -0.803 | 0.267 | 9.036 | 0.003 | 0.448 | 0.265-0.756 |
| 5-ASA (no) | 0.602 | 0.299 | 4.056 | 0.044 | 1.825 | 1.016-3.277 |
| Glucocorticoid (no) | -20.186 | 14748.005 | 0.000 | 0.999 | 0.000 | - |
| immunosuppressants (no) | 0.835 | 0.969 | 0.742 | 0.389 | 2.305 | 0.345-15.406 |
| biologics (no) | 0.525 | 0.931 | 0.318 | 0.573 | 1.691 | 0.273-10.476 |
| PLR | 0.017 | 0.003 | 43.439 | 0.000 | 1.018 | 1.012-1.023 |

B: regression coefficient; SEE: standard error of estimate; OR: odds ratio; CI: confidence interval; BMI: body mass index; 5-ASA: 5-aminosalicylic acid; PLR: platelet to lymphocyte ratio; gender (female), age, BMI, smoking (no), duration of disease, extent of colon (E3), 5-aminosalicylic acid (no), glucocorticoid (no), immunosuppressants (no), and biologics (no) were used as confounding factors, the categories analyzed are described with the reference category displayed in brackets.

Additional table 6. Binary multivariate logistic regression analyses of CAR and confounding factors in MES-ALL

| Variable | B | SEE | Wald | P value | OR | 95%CI |
|---|---|---|---|---|---|---|
| Age | -0.014 | 0.011 | 1.636 | 0.201 | 0.986 | 0.965-1.007 |
| Gender (female) | -0.001 | 0.322 | 0.000 | 0.998 | 0.999 | 0.531-1.880 |
| Smoke (no) | -0.264 | 0.429 | 0.380 | 0.537 | 0.768 | 0.331-1.779 |
| BMI | -0.011 | 0.038 | 0.089 | 0.765 | 0.989 | 0.917-1.065 |
| Duration | -0.003 | 0.002 | 1.980 | 0.159 | 0.997 | 0.993-1.001 |
| Extent (E3) | -0.217 | 0.277 | 0.612 | 0.434 | 0.805 | 0.467-1.387 |
| 5-ASA (no) | 0.229 | 0.309 | 0.550 | 0.458 | 1.258 | 0.686-2.305 |
| Glucocorticoid (no) | -19.312 | 12450.207 | 0.000 | 0.999 | 0.000 | - |
| immunosuppressants (no) | 0.070 | 0.974 | 0.005 | 0.943 | 1.072 | 0.159-7.237 |
| biologics (no) | 0.228 | 0.950 | 0.058 | 0.810 | 1.256 | 0.195-8.086 |
| CAR | 0.805 | 0.140 | 33.217 | 0.000 | 2.236 | 1.701-2.940 |

B: regression coefficient; SEE: standard error of estimate; OR: odds ratio; CI: confidence interval; BMI: body mass index; 5-ASA: 5-aminosalicylic acid; CAR: C-reactive protein to albumin ratio; gender (female), age, BMI, smoking (no), duration of disease, extent of colon (E3), 5-aminosalicylic acid (no), glucocorticoid (no), immunosuppressants (no), and biologics (no) were used as confounding factors, the categories analyzed are described with the reference category displayed in brackets.

Additional table 7. Binary multivariate logistic regression analyses of hs-CRP and confounding factors in MES-E3

| Variable | B | SEE | Wald | P value | OR | 95%CI |
|---|---|---|---|---|---|---|
| Age | -0.018 | 0.016 | 1.257 | 0.262 | 0.982 | 0.951-1.014 |
| Gender (female) | 0.340 | 0.549 | 0.383 | 0.536 | 1.404 | 0.479-4.115 |
| Smoke (no) | -0.334 | 0.673 | 0.246 | 0.620 | 0.716 | 0.192-2.677 |
| BMI | -0.103 | 0.063 | 2.733 | 0.098 | 0.902 | 0.798-1.019 |
| Duration | -0.008 | 0.004 | 4.363 | 0.037 | 0.992 | 0.985-1.000 |
| 5-ASA (no) | 0.052 | 0.484 | 0.012 | 0.914 | 1.053 | 0.408-2.718 |
| Glucocorticoid (no) | -17.025 | 23567.237 | 0.000 | 0.999 | 0.000 | - |
| immunosuppressants (no) | -0.990 | 1.444 | 0.470 | 0.493 | 0.372 | 0.022-6.301 |
| biologics (no) | 1.925 | 1.312 | 2.155 | 0.142 | 6.858 | 0.525-89.660 |
| Hs-CRP | 0.356 | 0.069 | 26.753 | 0.000 | 1.428 | 1.248-1.635 |

B: regression coefficient; SEE: standard error of estimate; OR: odds ratio; CI: confidence interval; BMI: body mass index; 5-ASA: 5-aminosalicylic acid; hs-CRP: high sensitivity C-reactive protein; gender (female), age, BMI, smoking (no), duration of disease, 5-aminosalicylic acid (no), glucocorticoid (no), immunosuppressants (no), and biologics (no) were used as confounding factors, the categories analyzed are described with the reference category displayed in brackets.

Additional table 8. Binary multivariate logistic regression analyses of MPV and confounding factors in MES-E3

| Variable | B | SEE | Wald | P value | OR | 95%CI |
|---|---|---|---|---|---|---|
| Age | -0.007 | 0.015 | 0.221 | 0.639 | 0.993 | 0.964-1.022 |
| Gender (female) | 0.560 | 0.476 | 1.382 | 0.240 | 1.750 | 0.688-4.451 |
| Smoke (no) | -0.385 | 0.563 | 0.466 | 0.495 | 0.681 | 0.226-2.054 |
| BMI | -0.081 | 0.056 | 2.092 | 0.148 | 0.922 | 0.826-1.029 |
| Duration | -0.006 | 0.003 | 3.676 | 0.055 | 0.994 | 0.989-1.000 |
| 5-ASA (no) | 0.517 | 0.438 | 1.393 | 0.238 | 1.677 | 0.711-3.960 |
| Glucocorticoid (no) | -20.296 | 25280.183 | 0.000 | 0.999 | 0.000 | - |
| immunosuppressants (no) | -1.660 | 1.679 | 0.976 | 0.323 | 0.190 | 0.007-5.115 |
| biologics (no) | 1.106 | 1.268 | 0.761 | 0.383 | 3.023 | 0.252-36.278 |
| MPV | -1.173 | 0.197 | 35.482 | 0.000 | 0.309 | 0.210-0.455 |

B: regression coefficient; SEE: standard error of estimate; OR: odds ratio; CI: confidence interval; BMI: body mass index; 5-ASA: 5-aminosalicylic acid; MPV: mean platelet volume; gender (female), age, BMI, smoking (no), duration of disease, 5-aminosalicylic acid (no), glucocorticoid (no), immunosuppressants (no), and biologics (no) were used as confounding factors, the categories analyzed are described with the reference category displayed in brackets.

Additional table 9. Binary multivariate logistic regression analyses of NLR and confounding factors in MES-E3

| Variable | B | SEE | Wald | P value | OR | 95%CI |
|---|---|---|---|---|---|---|
| Age | -0.001 | 0.013 | 0.011 | 0.916 | 0.999 | 0.973-1.025 |
| Gender (female) | 0.234 | 0.430 | 0.296 | 0.586 | 1.263 | 0.544-2.935 |

| | | | | | | |
|---|---|---|---|---|---|---|
| Smoke (no) | -0.278 | 0.548 | 0.258 | 0.612 | 0.757 | 0.259-2.216 |
| BMI | -0.116 | 0.053 | 4.813 | 0.028 | 0.891 | 0.803-0.988 |
| Duration | -0.007 | 0.003 | 7.458 | 0.006 | 0.993 | 0.988-0.998 |
| 5-ASA (no) | 0.406 | 0.399 | 1.034 | 0.309 | 1.501 | 0.686-3.281 |
| Glucocorticoid (no) | -19.612 | 25649.894 | 0.000 | 0.999 | 0.000 | - |
| immunosuppressants (no) | 0.089 | 1.188 | 0.006 | 0.940 | 1.093 | 0.107-11.220 |
| biologics (no) | 0.549 | 1.128 | 0.237 | 0.626 | 1.732 | 0.190-15.795 |
| NLR | 0.990 | 0.222 | 19.903 | 0.000 | 2.691 | 1.742-4.156 |

B: regression coefficient; SEE: standard error of estimate; OR: odds ratio; CI: confidence interval; BMI: body mass index; 5-ASA: 5-aminosalicylic acid; NLR: neutrophil to lymphocyte ratio; gender (female), age, BMI, smoking (no), duration of disease, 5-aminosalicylic acid (no), glucocorticoid (no), immunosuppressants (no), and biologics (no) were used as confounding factors, the categories analyzed are described with the reference category displayed in brackets.

Additional table 10. Binary multivariate logistic regression analyses of LMR and confounding factors in MES-E3

| Variable | B | SEE | Wald | P value | OR | 95%CI |
|---|---|---|---|---|---|---|
| Age | 0.003 | 0.014 | 0.056 | 0.814 | 1.003 | 0.976-1.031 |
| Gender (female) | 0.255 | 0.434 | 0.344 | 0.557 | 1.290 | 0.551-3.021 |
| Smoke (no) | 0.026 | 0.570 | 0.002 | 0.964 | 1.026 | 0.336-3.136 |
| BMI | -0.158 | 0.053 | 8.966 | 0.003 | 0.854 | 0.770-0.947 |
| Duration | -0.007 | 0.002 | 7.804 | 0.005 | 0.993 | 0.988-0.998 |
| 5-ASA (no) | 0.386 | 0.409 | 0.890 | 0.345 | 1.471 | 0.660-3.278 |
| Glucocorticoid (no) | -19.993 | 28410.669 | 0.000 | 0.999 | 0.000 | - |
| immunosuppressants (no) | -0.070 | 1.174 | 0.004 | 0.953 | 0.933 | 0.093-9.316 |
| biologics (no) | 0.545 | 1.264 | 0.186 | 0.666 | 1.725 | 0.145-20.530 |
| LMR | -0.578 | 0.115 | 25.243 | 0.000 | 0.561 | 0.448-0.703 |

B: regression coefficient; SEE: standard error of estimate; OR: odds ratio; CI: confidence interval; BMI: body mass index; 5-ASA: 5-aminosalicylic acid; LMR: lymphocyte to monocyte ratio; gender (female), age, BMI, smoking (no), duration of disease, 5-aminosalicylic acid (no), glucocorticoid (no), immunosuppressants (no), and biologics (no) were used as confounding factors, the categories analyzed are described with the reference category displayed in brackets.

Additional table 11. Binary multivariate logistic regression analyses of PLR and confounding factors in MES-E3

| Variable | B | SEE | Wald | P value | OR | 95%CI |
|---|---|---|---|---|---|---|
| Age | 0.006 | 0.014 | 0.228 | 0.633 | 1.007 | 0.980-1.034 |
| Gender (female) | 0.776 | 0.454 | 2.919 | 0.088 | 2.172 | 0.892-5.290 |
| Smoke (no) | -0.213 | 0.537 | 0.157 | 0.692 | 0.808 | 0.282-2.317 |
| BMI | -0.083 | 0.052 | 2.486 | 0.115 | 0.921 | 0.831-1.020 |
| Duration | -0.005 | 0.003 | 3.724 | 0.054 | 0.995 | 0.990-1.000 |
| 5-ASA (no) | 0.601 | 0.411 | 2.139 | 0.144 | 1.824 | 0.815-4.083 |

| | | | | | | |
|---|---|---|---|---|---|---|
| Glucocorticoid (no) | -20.620 | 27171.977 | 0.000 | 0.999 | 0.000 | - |
| immunosuppressants (no) | 0.064 | 1.252 | 0.003 | 0.959 | 1.066 | 0.092-12.418 |
| biologics (no) | 0.975 | 1.150 | 0.718 | 0.397 | 2.650 | 0.278-25.256 |
| PLR | 0.019 | 0.004 | 24.234 | 0.000 | 1.019 | 1.011-1.026 |

B: regression coefficient; SEE: standard error of estimate; OR: odds ratio; CI: confidence interval; BMI: body mass index; 5-ASA: 5-aminosalicylic acid; PLR: platelet to lymphocyte ratio; gender (female), age, BMI, smoking (no), duration of disease, 5-aminosalicylic acid (no), glucocorticoid (no), immunosuppressants (no), and biologics (no) were used as confounding factors, the categories analyzed are described with the reference category displayed in brackets.

Additional table 12. Binary multivariate logistic regression analyses of CAR and confounding factors in MES-E3

| Variable | B | SEE | Wald | P value | OR | 95%CI |
|---|---|---|---|---|---|---|
| Age | -0.021 | 0.016 | 1.732 | 0.188 | 0.979 | 0.948-1.010 |
| Gender (female) | 0.398 | 0.551 | 0.521 | 0.470 | 1.489 | 0.505-4.385 |
| Smoke (no) | -0.344 | 0.672 | 0.262 | 0.609 | 0.709 | 0.190-2.646 |
| BMI | -0.089 | 0.063 | 1.994 | 0.158 | 0.915 | 0.809-1.035 |
| Duration | -0.007 | 0.004 | 4.344 | 0.037 | 0.993 | 0.986-1.000 |
| 5-ASA (no) | 0.127 | 0.482 | 0.070 | 0.792 | 1.136 | 0.442-2.919 |
| Glucocorticoid (no) | -17.380 | 23446.071 | 0.000 | 0.999 | 0.000 | - |
| immunosuppressants (no) | -1.025 | 1.407 | 0.531 | 0.466 | 0.359 | 0.023-5.659 |
| biologics (no) | 1.912 | 1.355 | 1.991 | 0.158 | 6.766 | 0.475-96.311 |
| CAR | 1.246 | 0.244 | 26.073 | 0.000 | 3.478 | 2.155-5.612 |

B: regression coefficient; SEE: standard error of estimate; OR: odds ratio; CI: confidence interval; BMI: body mass index; 5-ASA: 5-aminosalicylic acid; CAR: C-reactive protein to albumin ratio; gender (female), age, BMI, smoking (no), duration of disease, 5-aminosalicylic acid (no), glucocorticoid (no), immunosuppressants (no), and biologics (no) were used as confounding factors, the categories analyzed are described with the reference category displayed in brackets.
